# S-nitrosoglutathione-loaded chitosan nanoparticles promote adaptive responses to water deficit in the critically endangered conifer *Araucaria angustifolia*

**DOI:** 10.3389/fpls.2026.1864816

**Published:** 2026-07-15

**Authors:** Rafael C. da Silva, Giovanna C. Do Carmo, Ana C. Preisler, Leonardo F. Tavares, Evandro A. Vieira, Joana C. Pieretti, Cecília B. Aragão, Amedea B. Seabra, Halley C. Oliveira, Marília Gaspar

**Affiliations:** 1 Department of Biodiversity Conservation, Institute of Environmental Research (IPA), São Paulo, SP, Brazil; 2 Postgraduate Program in Plant Biodiversity and Environment, Institute of Environmental Research (IPA), São Paulo, SP, Brazil; 3 Department of Animal and Plant Biology, State University of Londrina (UEL), Londrina, Paraná, Brazil; 4 Vale Institute of Technology (ITV), Belém, Brazil; 5 Center for Natural and Human Sciences (CCNH), Federal University of ABC (UFABC), Santo André, SP, Brazil

**Keywords:** abiotic stress tolerance, Brazilian pine, drought, GSNO, nanotechnology

## Abstract

*Araucaria angustifolia*, a conifer adapted to cold and high-altitude climates, is increasingly threatened by climate change, which further raises its existing risk of extinction. Projected changes in water availability are expected to expose this species to water deficit (WD), a condition to which it is highly susceptible. Thus, approaches aimed at developing strategies to enhance the tolerance of this species to WD are critically important. Nitric oxide (NO) is a gaseous signaling molecule that plays multiple roles in plant growth and development, particularly in responses to abiotic stress. Therefore, this study investigated the protective role of nanoencapsulated *S*-nitrosoglutathione (NP GSNO), an NO donor, in *A. angustifolia* subjected to WD. Seedlings of *A. angustifolia* grown under well-watered (WW) and WD conditions were treated with NP GSNO. Morphophysiological and biochemical parameters were assessed, including growth traits, stem water potential, oxidative stress markers, antioxidant enzyme activities, carbohydrate metabolism, and metabolic profiling. Results showed that NP GSNO treatment alleviated oxidative stress by enhancing antioxidant defenses, reducing ROS levels, and increasing RSNO content. Moreover, NP GSNO promoted the accumulation of osmoprotectants, as evidenced by increased soluble sugar levels, contributing to osmotic adjustment. Although WD reduced shoot and root growth, NP GSNO treatment mitigated these effects and promoted shoot growth. In conclusion, nanoencapsulated GSNO alleviated the deleterious effects of WD in *A. angustifolia* seedlings, through coordinated changes in redox balance, osmoprotective metabolism, and stress-associated metabolic pathways linked to drought acclimation.

## Introduction

As global warming intensifies, the frequency and severity of water scarcity events are expected to increase significantly (Seth and Sebastian, 2024). This trend poses a major challenge to the growth and survival of tree species, particularly some conifers, as it drives a gradual shift from mild- and cold-temperate trees to warm-temperate broad-leaved varieties. Consequently, an increase in temperature would result in a reduction in the dominance of conifers in their mild-temperate habitat and expansion of broad-leaved species (Fu et al., 2024). As outlined by the Intergovernmental Panel on Climate Change (IPCC, 2023) report, the projected intensification of global water scarcity due to climate change will impose significant limitations on ecosystem function. As a result, biodiversity will be severely impacted, with many plant species unable to adapt to the changing environmental conditions, thereby increasing their risk of extinction (Zhang et al., 2023).

In this context, *Araucaria angustifolia*, a critically endangered conifer native to South America, exemplifies the vulnerability of tree species to the escalating impacts of climate change (Thomas, 2013; Castro et al., 2020; Boonman et al., 2024). By the year 2070, *A. angustifolia* is projected to experience a 60% loss of its suitable climatic niches due to climate change (Castro et al., 2020). It is highly probable that suitable conditions will be limited to specific areas, such as high-elevation mountain ranges, underscoring the necessity for targeted conservation strategies to ensure the species’ survival (Castro et al., 2020). In addition, the decline in *A. angustifolia* populations not only threatens ecosystem functions but also highlights the broader risks faced by conifers in regions experiencing climatic shifts (Xu et al., 2023). While conifers, such as *A. angustifolia*, possess anatomical features that confer a certain degree of tolerance to water deficit (WD), such as needle-like leaves and a thick cuticle, these adaptations alone are often insufficient to withstand the increasing frequency and severity of prolonged drought events driven by climate change over the long term (Castro et al., 2020; Tagliari et al., 2021). Moreover, WD-induced hydraulic failure is a key mechanism of mortality in conifers, as prolonged WD increases xylem tension, promoting cavitation and embolism that disrupt water transport (Arend et al., 2021).

Water deficit refers to a condition in which plants experience insufficient water availability, leading to a decrease in soil water potential, which makes it more difficult for the plant to absorb water (Tardieu et al., 2011; Ahmad and Li, 2021). Such conditions can reduce plant growth and productivity, primarily by increasing root system lignification, and depressing root biomass accumulation, which in turn impair nutrient uptake and overall plant development (Bista et al., 2018; Ahmad and Li, 2021). Additionally, prolonged WD in plants can disrupt reactive oxygen species (ROS) homeostasis (Ahmad and Li, 2021). While ROS play a pivotal role in signaling under basal levels, elevated ROS levels can induce oxidative stress and cell damage, significantly impairing plant growth and development (Nadarajah, 2020).

To mitigate the harmful effects of increased ROS levels, plants have developed several mechanisms to maintain ROS homeostasis, such as the accumulation of antioxidant compounds and the activation of antioxidant enzymes (Dumanovic et al., 2021; Saini et al., 2024). Nitric oxide (NO), a gaseous reactive nitrogen species, plays a crucial role in plant physiology, by regulating gene expression and modulating protein activity through post-translational changes, such as *S*-nitrosylation (Astier et al., 2018; Wani et al., 2021; Saini et al., 2024). Additionally, NO mitigates ROS-induced damage by interacting with various signaling pathways, including those involving hormones and antioxidants (Wani et al., 2021; Saini et al., 2024). The protective effects of NO are particularly evident when applied exogenously, enhancing plant tolerance to abiotic stresses (da Silva et al., 2024; Saini et al., 2024). This is primarily achieved by increasing the activity of antioxidant enzymes, such as superoxide dismutase (SOD) and peroxidase (POD), which reduce oxidative damage, improve water retention, and promote root growth (da Silva et al., 2024; Saini et al., 2024).

Although NO provides important benefits to plants, its short half-life and instability restrict direct application (Bethke et al., 2004). Therefore, NO donors such as sodium nitroprusside (SNP), *S*-nitrosoglutathione (GSNO) and *S*-nitroso-L-cysteine (CySNO) are widely used (Floryszak‐Wieczorek et al., 2007). Recent studies demonstrate that these compounds enhance plant acclimation to abiotic stress. In drought-stressed common bean (*Phaseolus vulgaris*), SNP improved photosynthesis, antioxidant defense, nitrogen assimilation, proline metabolism and oxidative stress mitigation (Rehaman et al., 2025). Likewise, CySNO alleviated drought-induced oxidative damage in soybean (*Glycine max*) by strengthening antioxidant defenses, reducing electrolyte leakage and promoting root growth (Khan et al., 2026).

Among RSNOs, GSNO is particularly important as the main endogenous NO donor derived from glutathione *S*-nitrosylation, serving as a reservoir and transporter of NO in cells (Khator et al., 2024). Growing evidence highlights the effectiveness of GSNO in enhancing drought tolerance, improving gas exchange, osmotic adjustment, antioxidant metabolism, and redox balance in maize (*Zea mays)* (da Silva et al., 2025), as well as promoting adventitious rooting and stress acclimation in cucumber (*Cucumis sativus*) through modulation of ROS, the ascorbate–glutathione cycle, and protein S-nitrosylation (Hou et al., 2024). In addition, recent evidence indicates that the stress-mitigating effects of NO donors are closely associated with redox signaling networks involving ROS interactions, *S*-nitrosylation, and transcriptional regulation of stress-responsive pathways (Al Azzawi et al., 2026).

Despite their beneficial effects, the susceptibility of NO donors to environmental factors leads to their relatively rapid degradation and, thus, to reduced efficacy (Seabra and Durán, 2010; Seabra et al., 2014). In this context, nanotechnology offers a promising solution. By encapsulating NO donors in nanoparticles (NPs), it becomes possible to protect them from degradation, control their release, and enhance their overall efficacy (Lopes-Oliveira et al., 2019; do Carmo et al., 2021; Seabra et al., 2022; do Carmo et al., 2025; Gomes et al., 2025). One of the approaches employed is the incorporation of the NO donor into chitosan-based NPs, as chitosan is a biodegradable, biocompatible polymer that is widely employed as a nanocarrier system (Silveira et al., 2021; Seabra et al., 2022). This approach provides a cost-effective and efficient method for NO delivery through NO donors, with significant potential in agriculture and forestry (do Carmo et al., 2021; Silveira et al., 2021; Cabral et al., 2021; Seabra et al., 2022; Kondak et al., 2025).

Recent studies indicate that chitosan-based nanoparticles (NPs) loaded with NO donors effectively alleviate the effects of WD in plants (Silveira et al., 2019, Silveira et al., 2021; do Carmo et al., 2021; Methela et al., 2023a, Methela et al., 2023b; do Carmo et al., 2025). In sugarcane (*Saccharum* ssp.), nanoencapsulated NO donors induced stronger defense responses than free donors, likely due to improved control of NO release and protection against premature degradation (Silveira et al., 2019). Polymeric NPs may also enhance the absorption and distribution of bioactive molecules in plant tissues (Bombo et al., 2019; Takeshita et al., 2021). Consistently, do Carmo et al. (2021) showed that *S*-nitrosothiol nanoencapsulation increased RSNO levels in roots and leaves of *Heliocarpus popayanensis* and provided greater protection against WD than the free donor. Notably, only the nanoencapsulated formulation improved leaf relative water content and stimulated root hair formation, highlighting the potential of nanoparticle-assisted NO delivery to enhance stress acclimation.

In light of the intensifying effects of climate change, the reintroduction of native tree species through reforestation programs represents a crucial strategy for mitigating the global warming impact and the risk of extinction of threatened species (Nunes et al., 2020; Wang et al., 2024). Since WD stress poses significant challenges to the establishment of *A. angustifolia* seedlings, innovative technologies like nanoencapsulated NO donors may represent a promising avenue to improve the tolerance of these seedlings, and thus, contribute to their survival rate under these unfavorable conditions (Nabi et al., 2019; Preece et al., 2023; Saini et al., 2024). By utilizing techniques like nanoparticle-based smart delivery systems, essential compounds can be more effectively targeted, potentially improving plant tolerance to WD, salinity, and other harsh environmental conditions (Aguirre-Becerra et al., 2022).

The results presented by previous studies highlight the significant role played by NO donors in plants subjected to stress, underscoring the potential application of nanoencapsulated NO donors in producing more WD-tolerant tree seedlings. However, their effectiveness in addressing WD stress in highly susceptible and ecologically critical species, such as *A. angustifolia*, remains largely unexplored. Although NO-mediated responses to abiotic stress have been extensively investigated in angiosperms, considerably less is known regarding their roles in gymnosperms, particularly in endangered conifer species with distinct hydraulic and metabolic characteristics such as *A. angustifolia.* This critically endangered Brazilian native conifer plays a vital role in maintaining biodiversity and ecological stability within its native range (Thomas, 2013; Castro et al., 2020). Beyond its ecological significance, it is also of cultural and economic importance (Thomas, 2013; Castro et al., 2020).

Ensuring the survival and establishment of seedlings is crucial for the success of conservation and reforestation efforts (Preece et al., 2023). Thus, in the current study, we tested the hypothesis that GSNO-loaded chitosan nanoparticles would promote physiological and biochemical responses associated with improved acclimation to WD in *A. angustifolia* seedlings, including changes in antioxidant metabolism, osmotic adjustment, and stress-related metabolic pathways. By investigating these responses, we aim to provide insights into strategies that may contribute to seedling establishment and stress acclimation under adverse environmental conditions.

## Materials and methods

### Plant species and cultivation methods

The experiment was conducted in a greenhouse at the State University of Londrina (23°19’29”S, 51°11’51”W), where incident solar radiation was reduced by 55% using a shading structure. The light and temperature conditions were recorded by a thermohygrometer throughout the experiment. The experimental site is situated at an altitude of 586 meters under a humid subtropical climate (Cfa), with an average annual rainfall of 1400 mm and a mean annual temperature of 20 °C, as classified by Köppen (1948).

Seedlings of *A. angustifolia* (Bert.) Kuntze (Araucariaceae), approximately 1.5 years old and 38 cm in height, were cultivated in 1 L polystyrene pots filled with a commercial substrate containing limestone, Pinus/Eucalyptus bark, single superphosphate, and NPK (Mecplant, Telemaco Borba, Paraná, Brazil). Prior to the beginning of the experiments, all seedlings were maintained well-hydrated to ensure optimal growth.

### Synthesis of the formulations

Chitosan-based nanoparticles (NPs) were prepared using the ionotropic gelation method (Oliveira et al., 2016). A solution containing 2.6 mg mL^-^¹ of chitosan (low molecular weight, ≥75% deacetylated from Sigma-Aldrich (St. Louis, MO, USA) was dissolved in 1% acetic acid and mixed with reduced glutathione (GSH) at a concentration of 100 mM. The solution was mixed by magnetic stirring for 90 minutes, followed by the gradual addition of sodium tripolyphosphate (TPP) (0.6 mg mL^-^¹) in a 3:1 volumetric ratio of CS/GSH to TPP. The suspension was then stirred for an additional 45 minutes at room temperature to form NPs containing GSH.

To prepare the nanoparticles for plant application, sodium nitrite (NaNO_2_) was added to the suspension in an equimolar ratio to GSH. This step promoted the conversion of loaded GSH to GSNO by *S*-nitrosylation. The formation of GSNO-containing NPs was confirmed by the detection of an S-NO characteristic absorption band at 545 nm (Pelegrino et al., 2018). The resulting suspension was incubated in the dark at 10 °C for 1 hour and then diluted with distilled water to achieve the required GSNO concentrations.

### Characterization of the formulations

Nanoparticles were characterized by dynamic light scattering (DLS) and Nanoparticle Tracking Analysis (NTA), to determine the hydrodynamic size, zeta potential (ZP), and polydispersity index (PDI). DLS measurements were performed using a Zetasizer Nano ZS (Malvern Panalytical, UK) equipped with a DTS1070 cuvette. Analyses were conducted at 25 °C, using a 633 nm laser with a backscatter detection angle of 173°. Each sample was measured in triplicate to ensure reproducibility. The results were reported as the average hydrodynamic diameter, PDI, and ZP, with size distributions based on intensity to facilitate the identification of potential aggregates. Additionally, NTA was obtained using a NanoSight NS300 system equipped with a 532 nm laser (Malvern Instruments Co, Malvern, UK). The sample was prepared by diluting 15 µL of the NP suspension in 2 mL of water, injected into the equipment, and individually tracked. Results were automatically calculated using NTA 3.4 Build 3.4.4 software.

### Treatments

The seedlings were initially subjected to three treatments: control (water only), NPs containing 1 mM reduced glutathione (GSH), and NPs containing 1 mM *S*-nitrosoglutathione (GSNO). The NP GSH treatment was included as a non-nitrosylated control to evaluate the potential contributions associated with GSH and the nanocarrier. Because no substantial differences were observed between GSNO- and GSH-treated plants in the initial morphological analyses, the subsequent physiological and biochemical investigations focused on comparisons between the NP GSNO and control treatments.

The GSNO and GSH concentrations used in the present study were selected based on preliminary assays performed with concentrations commonly reported in the literature. As concentrations up to 100 µM did not promote any detectable growth responses in *A. angustifolia* seedlings (data not shown), the experiments of the present study were carried out with applications of GSNO in the millimolar range. However, the highest concentration tested (10 mM) caused deleterious effects on seedling growth (**Supplementary Table 1**) and thus only the 1 mM concentration was used in subsequent analyses. Moreover, dilution of the stock suspension resulted in an applied chitosan concentration of approximately 10 µg mL^-^¹, in addition to the 1 mM of GSNO.

At the start of the experiment, all plants received a single application in the soil of 80 mL of a solution containing either NPs with GSH, NPs with GSNO, or water alone. All treatments were subjected to two distinct water regimes: well-watered (WW) and water deficit (WD). In the WW treatment, pots were irrigated until free drainage and allowed to drain for 2 h to reach approximately 80% soil moisture. For the WD treatment, irrigation was gradually reduced until soil moisture reached approximately 30%, thereby avoiding abrupt dehydration and allowing progressive stress establishment. Pot weights were recorded daily, and soil moisture sensors (Echo EC5, HOBO), connected to a Micro Station Data Logger (H21, HOBO), were used to continuously monitor substrate moisture throughout the experiment (**Supplementary Figure 1**). Water was added as necessary to maintain the target moisture levels throughout the 30-day experimental period, ensuring stable and consistent WD conditions across treatments. The effectiveness of the imposed WD was further confirmed by the reductions in stem water potential observed at the end of the experiment.

### Morphophysiological analyses

The length of the shoots and primary roots was determined by direct measurement with a millimeter ruler. To obtain the dry weight (DW), the plant material was subjected to drying in an oven at 60 °C until a constant weight was achieved, which took approximately 72 hours. The total and specific lengths of the roots were determined using the gridline intersection method (Tennant, 1975), with adaptations to suit the specific requirements of this study. In summary, 100 mg of fresh root tissue was randomly positioned in a Petri dish with gridlines for the purpose of analyzing the intersection frequency. Subsequently, the resulting value was multiplied by the root dry weight and the constant 1.5. Additional root architectural traits, including lateral root number, average lateral root length, total and specific lateral root length, and lateral root density, were evaluated following the methodology described by da Silva et al. (2021).

Due to the needle-like morphology and reduced surface area of *A. angustifolia* leaves (**Supplementary Figure 2**), conventional infrared gas analyzer chambers are not suitable for reliable gas exchange measurements in this species. Therefore, stem water potential was used as the primary indicator of plant water status, determined by excising the stem from the plant and placing it in a Scholander pressure chamber. The pressure of the gas was increased gradually until evidence of sap flow was observed in the conductive tissue (Scholander et al., 1965).

### Biochemical analyses

All biochemical parameters were analyzed using fresh plant material, collected and immediately frozen in liquid nitrogen to preserve the biochemical integrity of the samples. Since WD substantially alters tissue hydration, the values obtained for biochemical analyses were adjusted using the fresh-to-dry mass ratio, to minimize the concentration effects associated with tissue dehydration and enable comparisons among treatments.

#### Oxidative stress markers

To quantify oxidative stress markers in plants, hydrogen peroxide (H_2_O_2_) and malondialdehyde (MDA) levels were measured in leaves and roots. A fresh portion of the plant tissue (50 mg) was collected and pulverized with 1.4 mL of 0.2% trichloroacetic acid (TCA) diluted in methanol. Subsequently, the supernatant was subjected to centrifugation at 13,700 x*g* for five minutes at 4 °C. The resulting supernatant was used for the analysis of MDA levels through the thiobarbituric acid reactive substances (TBARS) method (Camejo et al., 1998), and for the determination of H_2_O_2_ levels through the reaction with potassium iodide (KI) in phosphate buffer (Alexieva et al., 2001). The results of both analyses were obtained using a VICTOR3™ Multilabel Plate Reader (PerkinElmer Life and Analytical Sciences, Wallac, OY, Turku, Finland).

#### *S*-nitrosothiol (RSNO) quantification

The RSNO content in leaves and roots was evaluated as an indicator of NO bioavailability. Plant samples were homogenized with N-ethylmaleimide (NEM, 5 mM in PBS, pH 7.4) and subjected to sonication at 45 kHz for 10 minutes. Subsequently, the homogenate was subjected to centrifugation at 98,784 x*g* for 10 minutes. Next, 20 µL of the resulting supernatant were combined with 15 mL of copper chloride (CuCl_2_) solution (100 mM) to facilitate amperometric quantification of NO released from RSNO decomposition. Measurements were conducted using a WPI TBR4100/1025 free radical analyzer (World Precision Instruments Inc., Sarasota, FL, USA), which was equipped with an ISO-NOP sensor that was specific for NO (2 mm), as previously described by Oliveira et al. (2016). Subsequently, the data were compared to a standard curve generated with GSNO.

### Carbohydrate content

For total soluble sugars (TSS) quantification, one hundred milligrams of freeze-dried powdered leaves and roots were extracted in 1.5 mL of 80% ethanol at 80 °C for 15 minutes. The mixture was then centrifuged at 20,000 xg for 15 minutes at room temperature. The supernatant was collected, and the extraction was repeated four times. After the final extraction, the supernatants were combined, dried in a vacuum concentrator, and resuspended in 1 mL of water. Total soluble sugars were quantified by the phenol-sulfuric acid method on a spectrophotometer at 490 nm (Dubois et al., 1956).

#### Metabolic profile

Primary and secondary metabolites were extracted in methanol, chloroform, and water (2:1:2, v/v/v) with adonitol as internal standard, according to Lisec et al. (2006). After extraction, the samples were centrifuged at 2200 xg for 15 min, and 150 μL of the polar phase was transferred to a new tube and dried using a vacuum concentrator (SpeedVac). The precipitates were resuspended in 40 μL of pyridine solution and incubated under shaking at 37 °C for 2 hours. Subsequently, the samples were derivatized with N-methyl-N-trimethylsilyl trifluoroacetamide (MSTFA) for 30 minutes at 37 °C.

A volume of 1 μL of each sample was injected into a Shimadzu QP2020 gas chromatograph coupled to a mass spectrometer (GC-MS). Chromatograms and mass spectra were analyzed using the Labsolution software (Shimadzu) and metabolites were putatively annotated by comparing mass spectral fragmentation patterns with the NIST14 library and published literature. Only metabolites with a similarity index (SI) ≥ 80 were retained for further analyses. Metabolite annotation followed MSI confidence levels 2 and 3, depending on the degree of structural ambiguity among candidate compounds. Selected metabolites were analyzed using MetaboAnalyst 6.0 (https://www.metaboanalyst.ca). Metabolites with a VIP value >1 and p <0.05 were considered differentially expressed metabolites (DEMs). The expression pattern of the DEMs was presented in heatmaps, generated using the R statistic package.

#### Activity of antioxidant enzymes

Extracts were obtained by homogenizing 0.2 g of leaves and roots in 1 mL of extraction buffer (1 mM EDTA, 0.1 M potassium phosphate buffer, pH 7.5, 2% (w/v) polyvinylpolypyrrolidone, 3 mM DTT, and 5 mM ascorbate). The homogenate was centrifuged at 15,645 x*g* at 4 °C for 20 minutes. Superoxide dismutase activity (SOD, EC 1.15.1.1) was determined according to Giannopolitis and Ries (1977), by measuring the ability of the extract to inhibit the photoreduction of nitro blue tetrazolium chloride (NBT). One unit of SOD activity was defined as the enzyme activity required to inhibit the photoreduction of NBT by 50% compared to the control. Peroxidase activity (POD, EC 1.11.1.7) was determined according to Peixoto et al. (1999), by monitoring the increase in absorbance at 420 nm due to the oxidation of pyrogallol in the presence of H_2_O_2_. One unit of POD activity was defined as the amount of enzyme responsible for the formation of 1 μmol of purpurogallin per minute under the assay conditions, calculated using the molar extinction coefficient of purpurogallin (ϵ = 2.47 mM^-^¹ cm^-^¹). Specific enzyme activities were expressed as enzyme units per milligram of protein. Protein content in the extracts was quantified using Bradford reagent (Bio-Rad), following the manufacturer’s instructions. Absorbance was measured at 595 nm using an Agilent BioTek Epoch microplate spectrophotometer.

### Statistical analyses

The treatments were arranged in a completely randomized experimental design. Ten to twelve replicates were used for morphological analyses, and five replicates were used for biochemical analyses. When the data met the assumptions (normality and homogeneity of variance), they were subjected to a two-way analysis of variance (ANOVA, water condition x formulation treatment). When significant, means were compared using the Tukey test (*p* < 0.05). The metabolic dataset was subjected to hierarchical clustering heatmap analysis and enrichment analysis (Over Representation Analysis – ORA), to identifying the major classes and subclasses of the detected metabolites. The metabolic data were analyzed using the free web-based metabolomics tool MetaboAnalyst 6.0 (https://www.metaboanalyst.ca).

## Results

**Figure 1:**
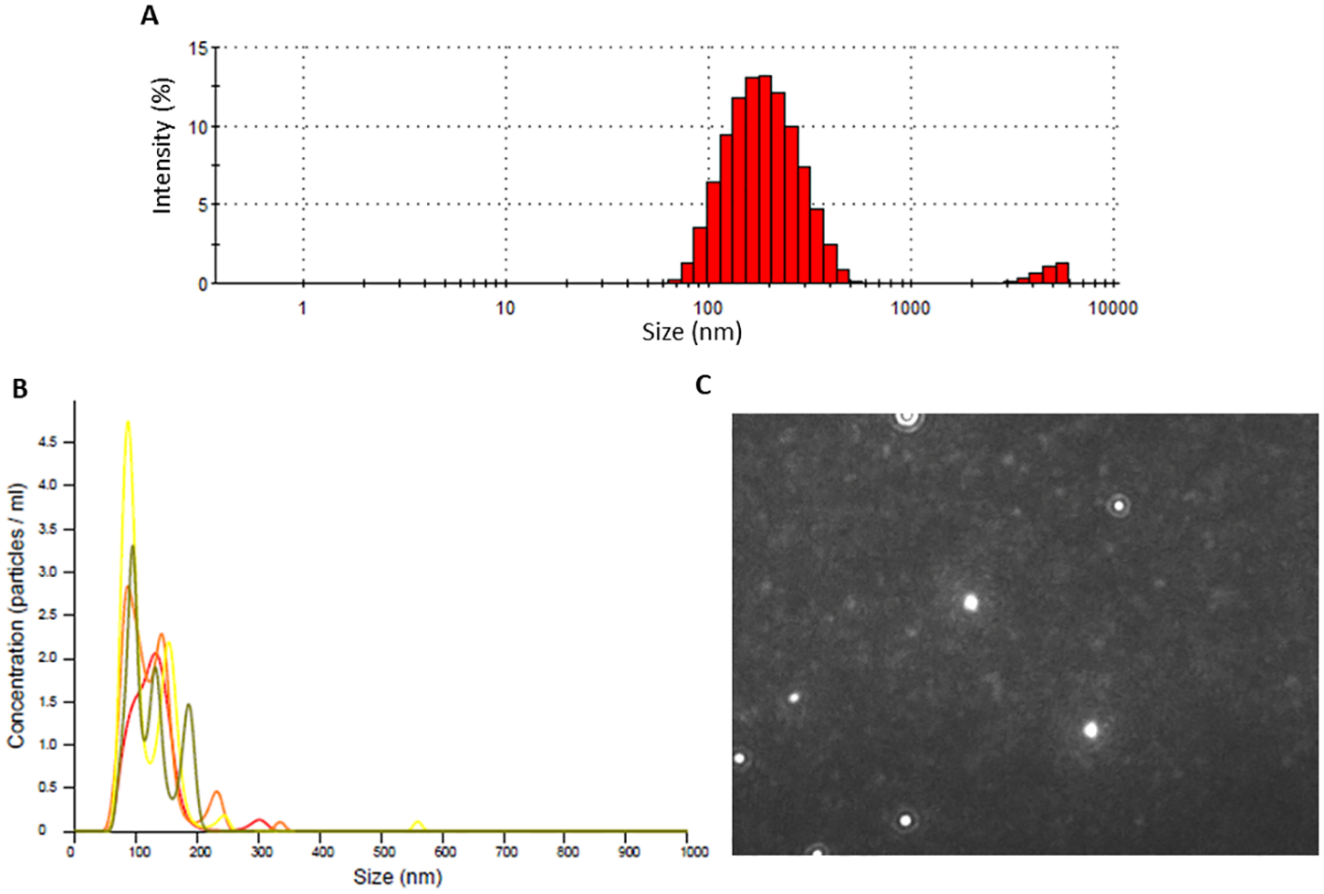
Hydrodynamic size characterization of the synthesized nanoparticles by **(A)** Dynamic Light Scattering (DLS) intensity-weighted size distribution, **(B)** Nanoparticle Tracking Analysis (NTA) size distribution by concentration across quadruplicate measurements, and **(C)** Representative NTA video frame showing the light scattering of individual nanoparticles undergoing Brownian motion.

Physicochemical characterization confirmed the successful formation of NPs containing a high concentration of GSH (100 mM). According to DLS analysis (**Figure 1A**), the NPs exhibited a hydrodynamic size of 170.5 ± 7.7 nm, PDI of 0.27 ± 0.05, and positive ZP of +17.5 ± 1.5 mV. The data obtained by NTA, presented in **Figure 1B**, corroborated those of DLS, indicating a mean size of 123.4 ± 1.9 nm. The real-time visualization of individual nanoparticles undergoing Brownian motion is depicted in **Figure 1C**. The complete dataset can be found in the **Supplementary Material**, demonstrating the batch-to-batch reproducibility of the synthesis protocol (**Supplementary Table 1**). Additionally, NTA revealed a particle concentration of 2.01 × 10^9^ particles mL^-1^ in the suspension.

**Figure 2:**
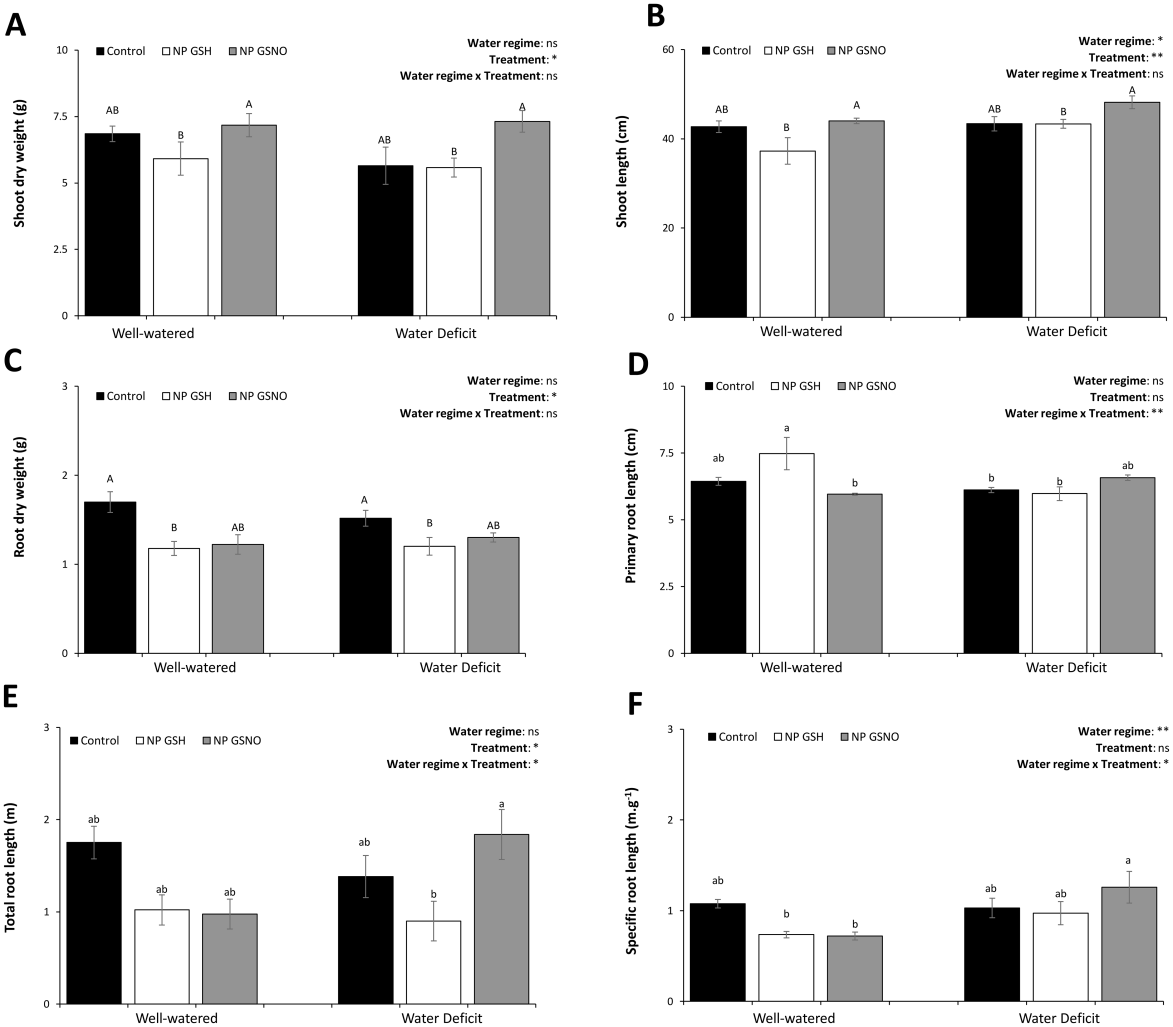
Effects of water regime and nanoencapsulated GSNO treatment on **(A)** shoot dry weight, **(B)** shoot length, **(C)** root dry weight, **(D)** primary root length, **(E)** total root length, and **(F)** specific root length. Plants were grown under well-watered or water-deficit conditions and treated with either NPs containing GSNO, NPs containing GSH (non-nitrosylated control), or water-only as a control. Black bars represent control plants, white bars represent NP GSH-treated plants, and gray bars represent NP GSNO-treated plants. Values are means ± standard error (SE). Different lowercase letters indicate significant differences among treatment × water-regime combinations, while different uppercase letters indicate significant main effects of treatment within each water regime, as determined by two-way ANOVA followed by *post hoc* tests (p < 0.05). Significance of main effects and interactions is denoted as ***p < 0.001, **p < 0.01, *p < 0.05, and ns, not significant.

To evaluate whether NP GSNO and NP GSH application influenced plant performance, morphological parameters were first assessed under both water regimes. The application of GSNO-loaded nanoparticles resulted in significantly greater shoot dry weight and length values compared with GSH-treated plants under both WW and WD conditions (**Figures 2A, B**). Under WD, NP GSNO-treated plants also exhibited the highest total root length, which differed statistically from the non-nitrosylated NP GSH treatment (**Figure 2E**).

Plants subjected to the WD regime did not differ in shoot length or shoot dry weight compared with WW plants (**Figure 2A**). In contrast, NP GSH-treated plants showed reduced root dry weight relative to the control under both WW and WD conditions, while NP GSNO-treated plants exhibited intermediate values for this parameter. For primary root length, NP GSH-treated plants under WW displayed the highest mean values, although these did not differ statistically from the WW control. Under WD, no significant differences were observed among treatments. Regarding total root length, WD plants treated with NP GSNO showed greater values than those receiving NP GSH, with control plants again presenting intermediate responses. A similar pattern was observed for specific root length, where NP GSNO tended to increase values under WD conditions, although the differences were not statistically significant (**Figures 2E, F**).

The NP GSNO treatment also promoted a higher lateral root number, and thus a higher lateral root density relative to NP GSH-treated seedlings under WD conditions (**Supplementary Figure 3**). No other root architecture parameters exhibited significant differences among treatments under either WW or WD conditions. Additionally, NP GSNO at 1 mM promoted more favorable responses under WD conditions, including lateral root number, total lateral root length, and specific lateral root length compared with the WD control plants (**Supplementary Table 2**). In contrast, the highest concentration tested (10 mM) failed to improve these parameters and, for some root-related traits, resulted in lower values than those observed in either WD control or the NP GSNO 1 mM treatments (**Supplementary Table 2**). Under WD, seedlings treated with NP GSNO 10 mM exhibited marked reductions in lateral root number, total root length, and total lateral root length.

**Figure 3:**
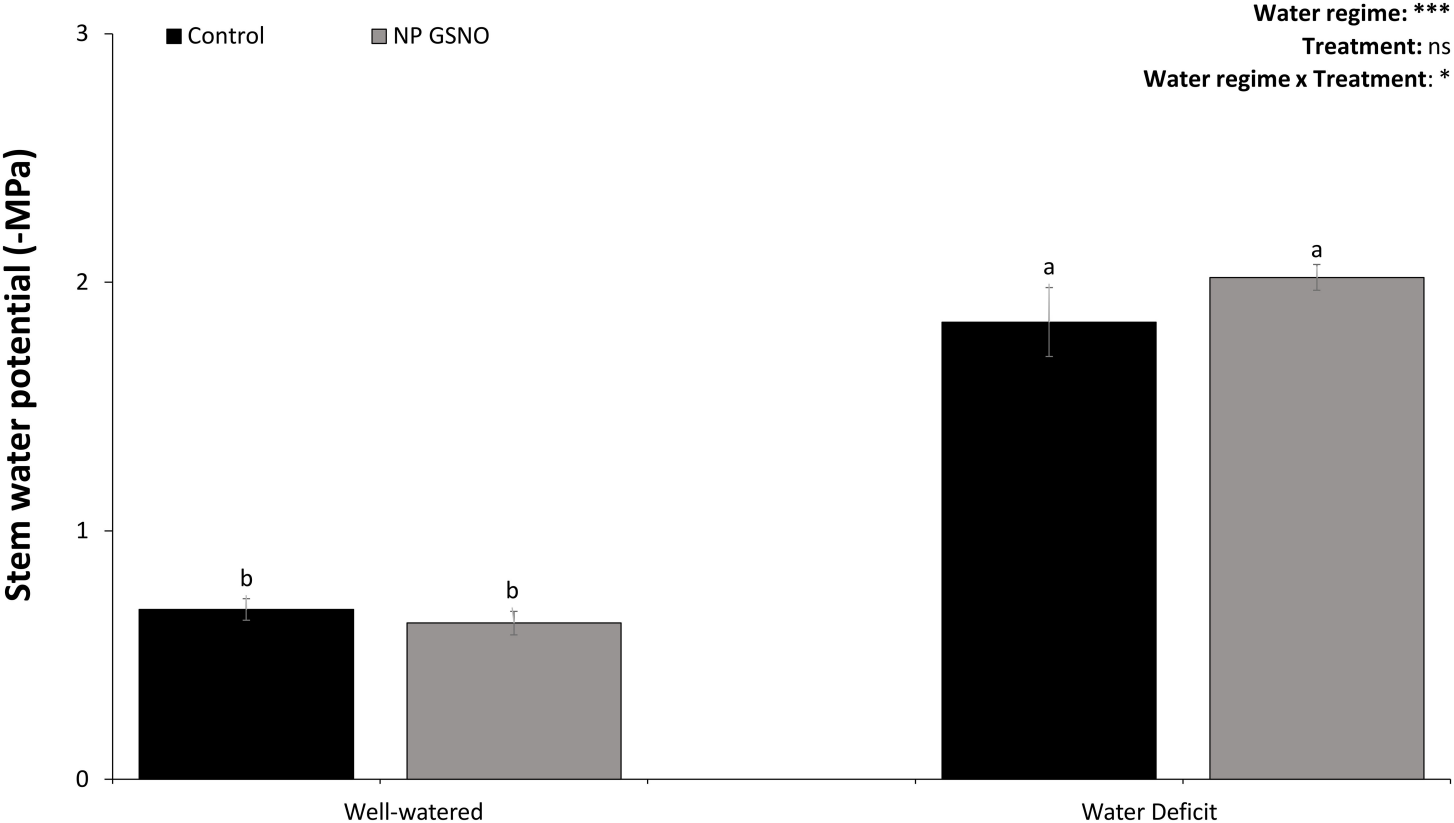
Effects of water regime and nanoencapsulated GSNO treatment on stem water potential. Plants were grown under well-watered or water-deficit conditions and treated with NPs containing GSNO (gray bars) or water-only as a control (black bars). Values are means ± standard error (SE). Different lowercase letters indicate significant differences among treatment × water-regime combinations, while different uppercase letters indicate significant main effects of treatment within each water regime, as determined by two-way ANOVA followed by *post hoc* tests (p < 0.05). Significance of main effects and interactions is denoted as ***p < 0.001, **p < 0.01, *p < 0.05, and ns, not significant.

To determine whether these growth responses were associated with changes in plant water status, stem water potential was evaluated. Consistent with the imposed water regimes, plants subjected to WD exhibited a markedly more negative stem water potential, indicative of the physiological stress induced by limited water availability (**Figure 3**). However, no statistically significant differences were found among treatments.

**Figure 4:**
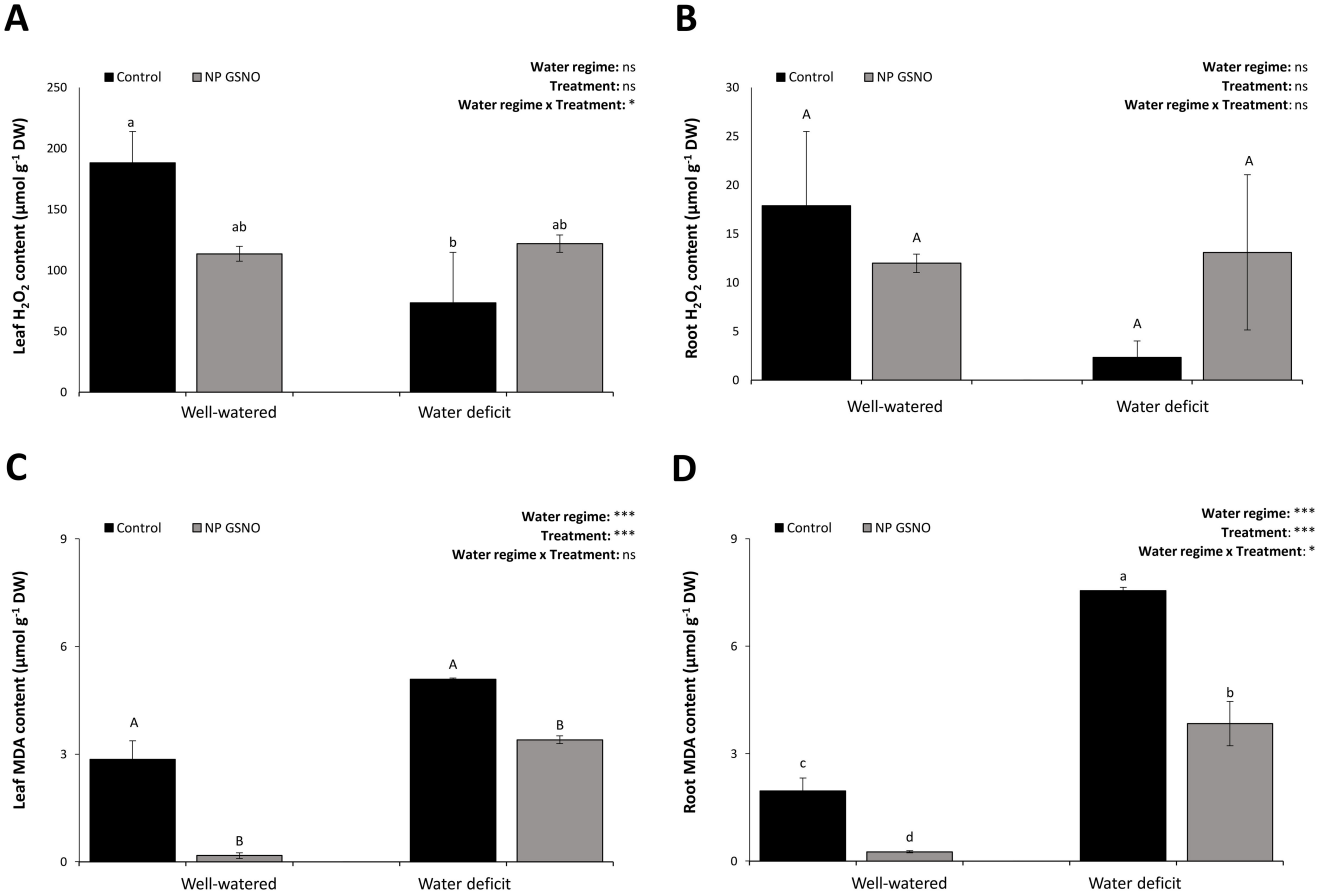
Effects of water regime and nanoencapsulated GSNO treatment on **(A)** leaf and **(B)** root hydrogen peroxide (H_2_O_2_) content, as well as **(C)** leaf and **(D)** root malondialdehyde (MDA) content. Plants were grown under well-watered or water-deficit conditions and treated with NPs containing GSNO (gray bars) or water-only as a control (black bars). Values are means ± standard error (SE). Different lowercase letters indicate significant differences among treatment × water-regime combinations, while different uppercase letters indicate significant main effects of treatment within each water regime, as determined by two-way ANOVA followed by *post hoc* tests (p < 0.05). Significance of main effects and interactions is denoted as ***p < 0.001, **p < 0.01, *p < 0.05, and ns, not significant.

To examine whether these conditions affected cellular redox homeostasis, H_2_O_2_ and MDA levels were quantified. H_2_O_2_ content decreased in the leaves of control plants under WD compared with WW controls, whereas in NP GSNO-treated plants the content remained unchanged under both water regimes (**Figure 4A**). In roots, H_2_O_2_ levels showed a similar profile, with no significant differences among water regimes or treatments (**Figure 4B**). In contrast, MDA content, an indicator of lipid peroxidation, was significantly reduced by NP GSNO application in both leaves and roots under WW and WD conditions (**Figures 4C, D**).

**Figure 5:**
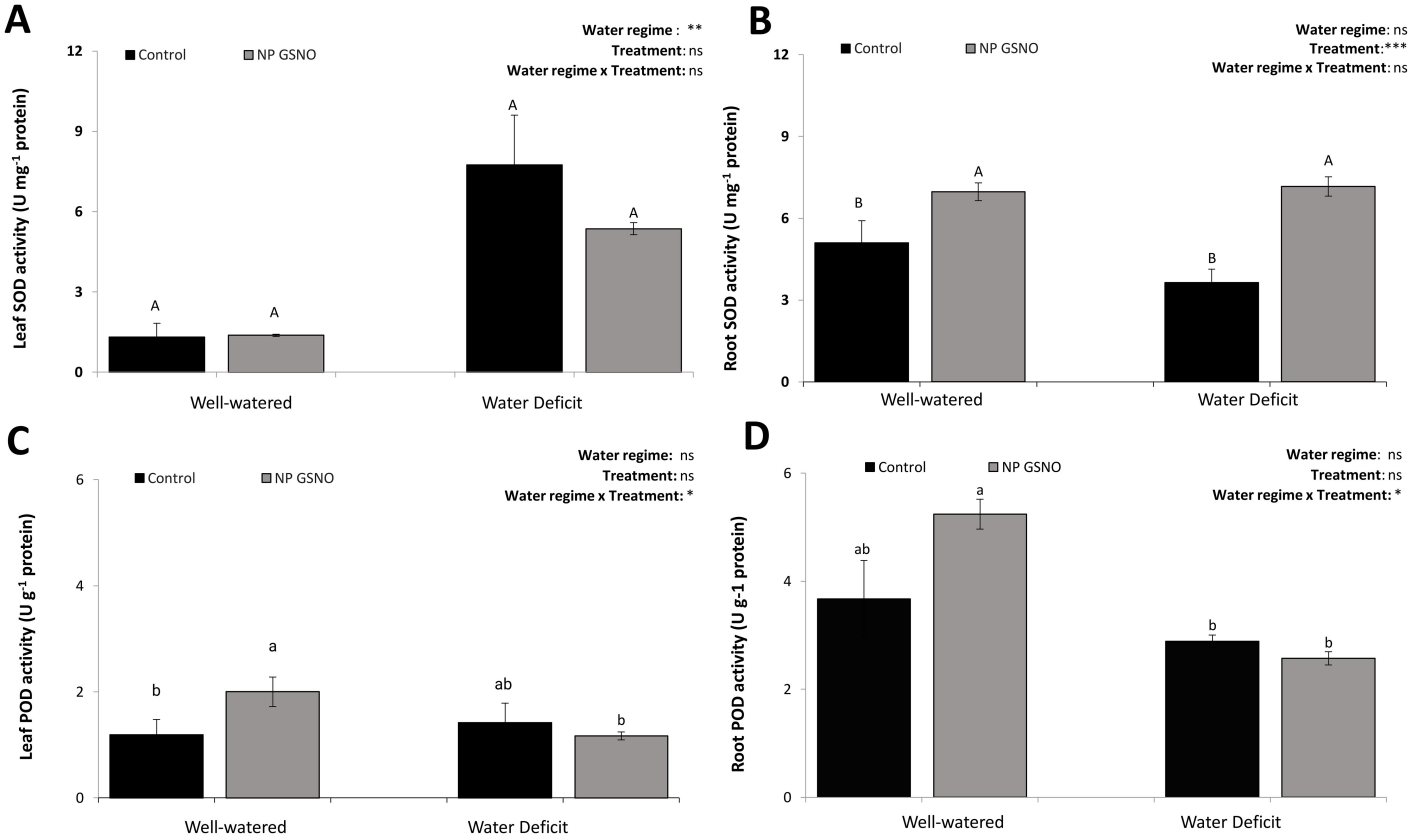
Effects of water regime and nanoencapsulated GSNO treatment on **(A)** leaf and **(B)** root superoxide dismutase (SOD) activity, as well as **(C)** leaf and **(D)** root peroxidase (POD) activity. Plants were grown under well-watered or water-deficit conditions and treated with NPs containing GSNO (gray bars) or water-only as a control (black bars). Values are means ± standard error (SE). Different lowercase letters indicate significant differences among treatment × water-regime combinations, while different uppercase letters indicate significant main effects of treatment within each water regime, as determined by two-way ANOVA followed by *post hoc* tests (p < 0.05). Significance of main effects and interactions is denoted as ***p < 0.001, **p < 0.01, *p < 0.05, and ns, not significant.

The activity of antioxidant enzymes was subsequently analyzed, revealing that NP GSNO application altered antioxidant responses (**Figure 5**). Although SOD activity did not differ among treatments in leaves (showing variation only between the WW and WD regimes), in roots, NP GSNO treatment increased SOD activity under both water regimes (**Figures 5A, B**). Regarding POD, NP GSNO application significantly enhanced its activity in the leaves of plants under WW conditions, whereas no differences from the control were observed under WD. A similar pattern was found in roots, where NP GSNO increased POD activity in WW plants but did not alter enzyme activity under WD conditions (**Figures 5C, D**).

**Figure 6:**
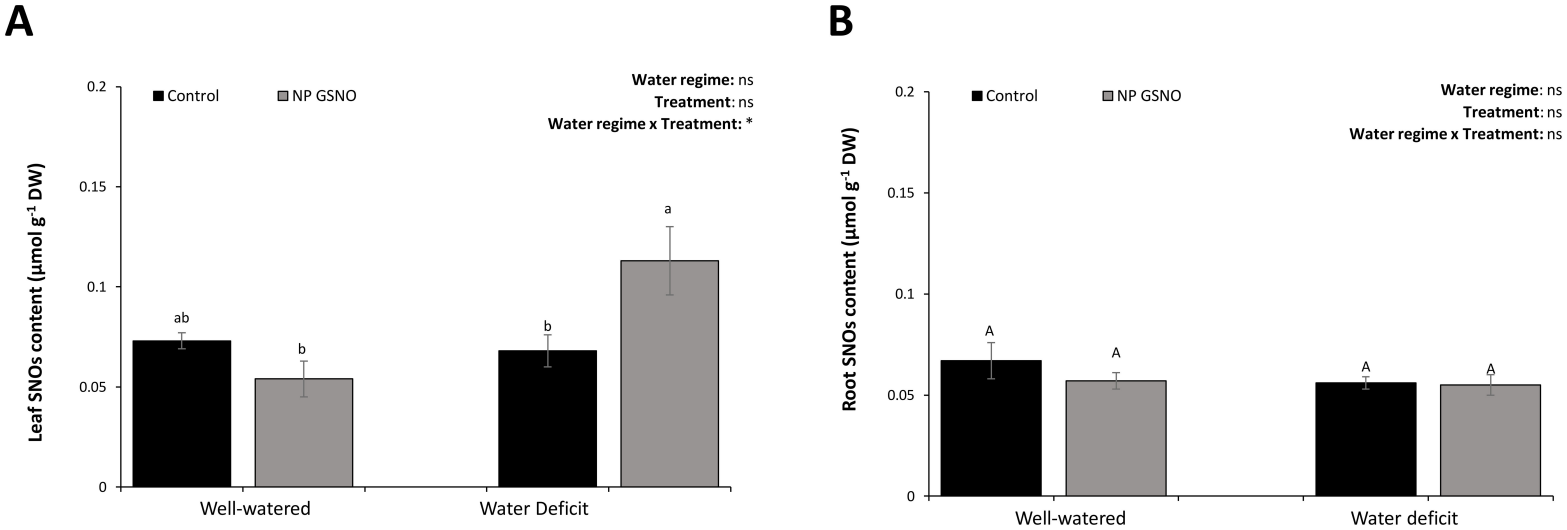
Effects of water regime and nanoencapsulated GSNO treatment on **(A)** leaf and **(B)** root *S*-nitrosothiol (RSNO) content. Plants were grown under well-watered or water-deficit conditions and treated with NPs containing GSNO (gray bars) or water-only as a control (black bars). Values are means ± standard error (SE). Different lowercase letters indicate significant differences among treatment × water-regime combinations, while different uppercase letters indicate significant main effects of treatment within each water regime, as determined by two-way ANOVA followed by *post hoc* tests (p < 0.05). Significance of main effects and interactions is denoted as ***p < 0.001, **p < 0.01, *p < 0.05, and ns, not significant.

Given that GSNO is an NO donor, endogenous RSNO levels were determined next, to assess possible NO-related signaling effects. The application of NP GSNO significantly increased levels in leaves of WD plants compared to the control (**Figure 6A**). In contrast, in WW plants, no differences were observed following NP GSNO application. Similarly, no significant differences were observed in the roots when comparing the different water regimes and NP treatments (**Figure 6B**).

**Figure 7:**
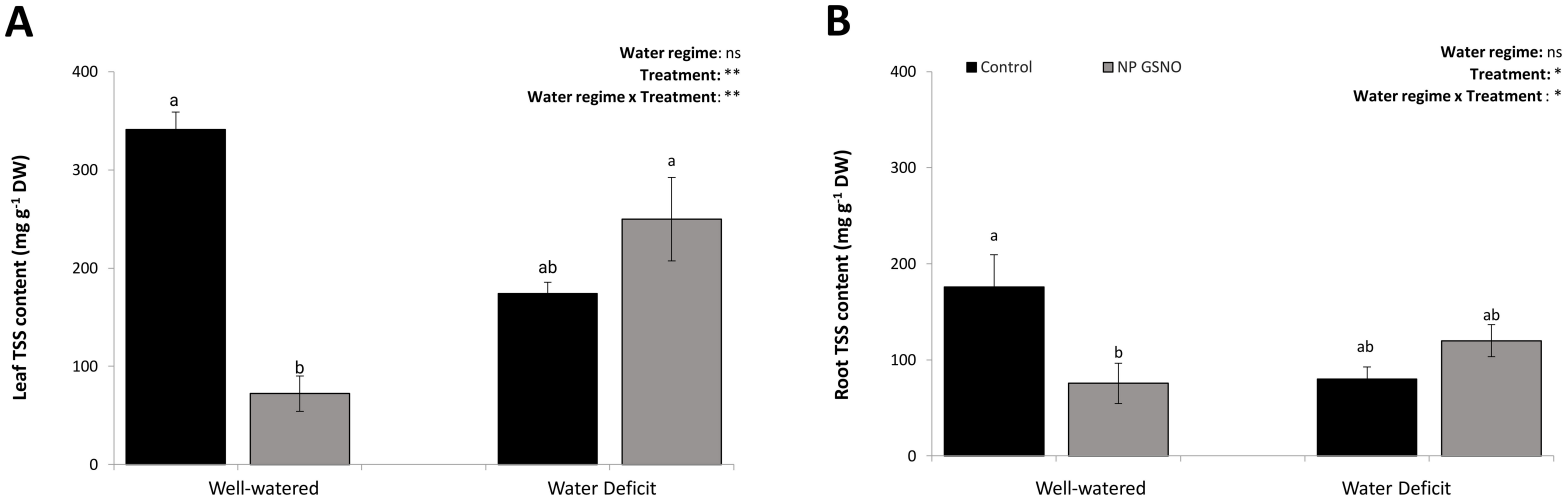
Effects of water regime and nanoencapsulated GSNO treatment on **(A)** leaf and **(B)** root total soluble sugar content. Plants were grown under well-watered or water-deficit conditions and treated with NPs containing GSNO (gray bars) or water-only as a control (black bars). Values are means ± standard error (SE). Different lowercase letters indicate significant differences among treatment × water-regime combinations, while different uppercase letters indicate significant main effects of treatment within each water regime, as determined by two-way ANOVA followed by *post hoc* tests (p < 0.05). Significance of main effects and interactions is denoted as ***p < 0.001, **p < 0.01, *p < 0.05, and ns, not significant.

Metabolic responses associated with stress tolerance were further explored through soluble sugar quantification. Although NP GSNO application led to a decrease in TSS values in leaves of WW plants, the WD plants showed an opposite response, with an increase in TSS content (**Figure 7A**).

**Figure 8:**
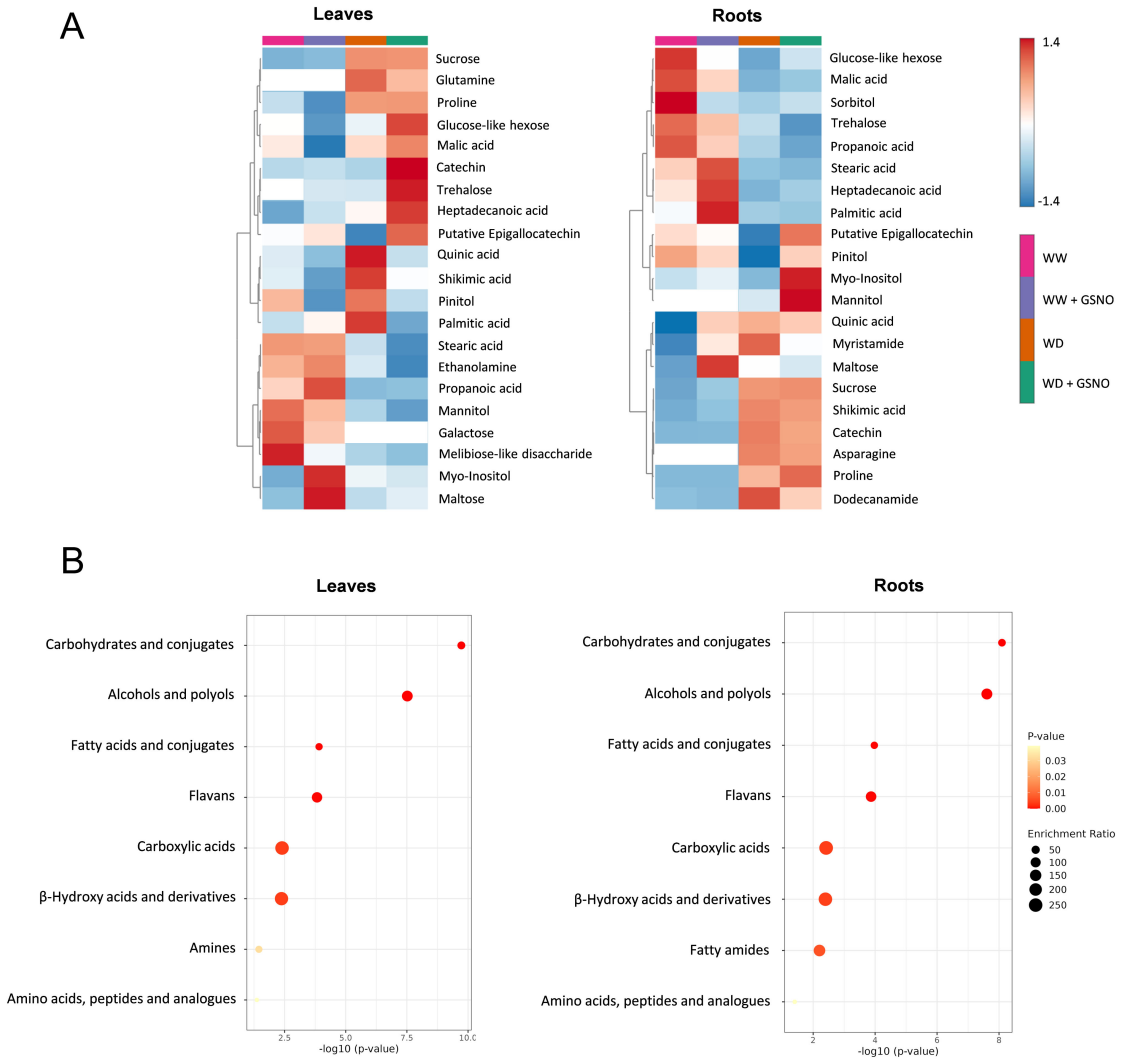
Metabolic responses of *A. angustifolia* seedlings subjected to well-watered (WW) and water deficit (WD) conditions with or without NP GSNO treatment. **(A)** Heatmap of differentially expressed metabolites (DEMs) identified in leaves and roots. Metabolites were selected based on VIP > 1 and p < 0.05. Colors indicate relative metabolite abundance after autoscaling. **(B)** Over-representation analysis (ORA) showing the major metabolite classes enriched in leaves and roots. Dot size represents enrichment ratio, whereas color intensity indicates statistical significance (-log10 p-value).

A metabolic profiling analysis was carried out. Twenty-one differential expressed metabolites (DEM) were detected in both leaves and roots after 30 days of the experiment (**Figures 8A, B**).

The identified DEM in leaves and/or roots include three amino acids, six carbohydrates (non-structural), four sugar alcohols (polyols), four organic acids, four fatty acids, two phenolic compounds, two fatty amides and one amino alcohol (ethanolamine). In leaves of WW plants, higher relative accumulation of maltose, galactose and mannitol was observed, whereas leaves of WD plants showed increased relative abundance of sucrose, regardless of the presence of NP GSNO. Notably, NP GSNO application maintained or enhanced the accumulation of glucose, sucrose and trehalose in WD leaves. In roots, differential accumulation was also detected, including some of the aforementioned sugars as well as the polyols myo-inositol, pinitol and mannitol. Similarly, regardless of NP GSNO application, amino acids such as glutamine and proline accumulated in leaves under WD conditions, while asparagine accumulated in roots under the same treatments (**Figure 8A**).

With regard to foliar organic and fatty acids, malic acid increased substantially in both WD treatments, whereas palmitic, quinic, and shikimic acids were more abundant under WD conditions. In contrast, heptadecanoic acid exhibited higher accumulation in WD + NP GSNO. In roots, organic acids were generally less abundant, although shikimic acid increased in both WD treatments. Under WD conditions, NP GSNO application led to the accumulation of myristamide and dodecanamide, indicating alterations in lipid-related metabolism. The marked increase in shikimic acid in both tissues likely contributed to enhanced biosynthesis and accumulation of phenolic compounds. This is supported by substantial increases in levels of catechin in roots under both WD and WD + GSNO, as well as epigallocatechin in leaves and roots exclusively under the WD + GSNO treatment (**Figure 8A**).

The main classes of compounds accumulated in leaves and roots of *A*. *angustifolia* included carbohydrates and their conjugates, alcohols and polyols, fatty acids and their conjugates, flavans, carboxylic acids, β-hydroxy acids, amines and amino acids and peptides (**Figure 8B**). The accumulation of these metabolites highlights the key metabolic pathways associated with plant hydration status and NP GSNO treatment. While WW plants exhibited higher relative abundance of metabolites associated with primary carbon metabolism, plants under WD treatment exhibited metabolic adjustments to cope with stress. As shown in **Supplementary Figure 4**, NP GSNO-induced modulation of the shikimate and phenylpropanoid pathways constitutes an important mechanism underlying WD acclimation.

## Discussion

### Hydraulic vulnerability and water deficit acclimation responses of Araucariaceae species under water deficit

The responses observed in *A. angustifolia* seedlings under WD conditions are consistent with previous reports describing the WD sensitivity of Araucariaceae species. In the present study, WD conditions resulted in stem water potential values close to −2 MPa, indicating substantial effects on plant water status. Although lethal water potential thresholds for *A. angustifolia* seedlings remain poorly characterized, previous studies on conifers showed that Araucariaceae species exhibit relatively vulnerable hydraulic systems and lower dehydration tolerance compared with several other conifer lineages under WD conditions (Brodribb et al., 2014). Therefore, the water potential values observed in the present study likely represent physiologically relevant stress conditions for this species.

Papú et al. (2021), evaluating *Araucaria araucana* seedlings under water restriction, observed reductions in biomass accumulation, together with increased proline accumulation, stem wood density, and mortality under prolonged drought. The authors concluded that *A. araucana* seedlings can tolerate short-term drought through physiological acclimation responses, whereas more severe or prolonged dehydration strongly compromises seedling performance and survival. These findings are consistent with the present study, in which *A. angustifolia* seedlings exhibited clear biochemical and metabolic responses to WD even in the absence of pronounced reductions in shoot biomass. Studies comparing conifer lineages have further demonstrated that Araucariaceae species generally exhibit conservative drought-response strategies and high sensitivity to hydraulic dysfunction under water deficit conditions.

Zimmer et al. (2016) showed that several Araucariaceae species display strong isohydric behavior, maintaining relatively stable leaf water potential during dehydration, through rapid stomatal closure, but at the cost of reduced gas exchange and high drought sensitivity. Similarly, Brodribb et al. (2014), evaluating 42 conifer species, identified Araucariaceae as one of the least WD-tolerant conifer groups, characterized by lower tolerance to extreme water deficit compared with several Cupressaceae species. According to these authors, Araucariaceae species rely predominantly on conservative water-use strategies involving ABA-mediated stomatal closure during WD, rather than on highly cavitation-resistant xylem systems. Together, these studies indicate that Araucariaceae species generally exhibit pronounced physiological and metabolic adjustments under WD conditions, particularly involving osmotic regulation, oxidative stress responses, and growth modulation. This interpretation is consistent with the present study, in which *A. angustifolia* seedlings subjected to WD exhibited substantial changes in antioxidant metabolism, osmoprotective compounds, and root-related traits, even in the absence of marked reductions in shoot biomass accumulation.

### Physicochemical performance of GSNO-loaded chitosan nanoparticles and relevance for plant delivery

The treatment with chitosan nanoparticles loaded with the NO donor (GSNO) demonstrated potential for promoting adaptative responses in *A. angustifolia* growth under different conditions. The synthesis of chitosan nanoparticles via the ionotropic gelation method is well established in the literature (Oliveira et al., 2016; Silveira et al., 2021; Gomes et al., 2025), being based on the electrostatic interaction between the positively charged groups of chitosan and the negatively charged phosphates of tripolyphosphate (TPP). The positive zeta potential (+14.4 ± 2.7 mV) observed in the current study is a characteristic indicator of the presence of chitosan on the nanoparticle surface. This reflects the cationic nature of the protonated amino groups within the polymeric chain, which is consistent with our previous findings for chitosan NPs encapsulating NO donors (Silveira et al., 2019, Silveira et al., 2021). Regarding particle size and distribution, the hydrodynamic size values evaluated by DLS (170.5 ± 7.7 nm) confirmed the nanometric scale, while the PDI (0.27 ± 0.05) indicated a moderate polydispersity index, similar to other studies utilizing chitosan NPs (Silveira et al., 2021; Korpayev et al., 2021). Notably, the low standard deviations obtained from four independent batches prepared over multiple months provide strong physical evidence of the high reproducibility of the synthesis protocol. The size discrepancy observed between DLS and NTA (123.1 nm) is a common analytical occurrence and corroborates the dynamic profiles of chitosan NPs. By utilizing Brownian motion to determine the number and size of individual particles, NTA offers higher resolving power for polydisperse samples (Wells et al., 2024). Recent studies using NTA to characterize chitosan NPs have reported concentrations in the order of 3.29 × 10^9^ nanoparticles mL^-1^ and identified multiple size populations (with means ranging from 89 nm to 139 nm) within the same sample (Gomes et al., 2025). Thus, the diameter obtained via NTA (123.1 nm) for the formulation, as well as the concentration of 2.01 × 10^9^ particles/mL^-1^, provide robust physical evidence of both the synthesis quality and the morphological consistency of the chitosan NPs with parameters described in the literature, which typically range between 120 nm and 167 nm depending on the applied conditions and dilutions (Silveira et al., 2021; da Veiga et al., 2024). Therefore, our synthesis pathway demonstrates a prominent pathway to use in plants.

It is worth mentioning that, in the present study, the concentration of GSH in the synthesized nanoformulation (100 mM) is higher than that reported in previous studies (*e.g.*, 1–40 mM; Silveira et al., 2019, Silveira et al., 2021). Prior to plant application, the stock suspension was diluted to the final concentration used in the treatments, as described in the Material and Methods section. The use of a more concentrated formulation is particularly important for experiments involving species that require relatively high concentrations of NO donors to elicit protective responses, as observed for *A. angustifolia*, in which the beneficial effects were detected at 1 mM of GSNO.

### Nanoencapsulated GSNO modulates oxidative and metabolic responses under water deficit

There is a notable gap in studies examining the impact of WD on the morphophysiological and biochemical responses of *A. angustifolia* seedlings, thereby underscoring the need to investigate the responses of this critically endangered conifer within the context of climate change. The application of NP GSNO resulted in a reduction in MDA content in the leaves and roots of the plants, suggesting a potential role for the nanoencapsulated NO donor in mitigating oxidative damage regardless of the irrigation regime. However, under WD, NP GSNO-treated plants tended to show higher H_2_O_2_ levels than control plants. This apparent disconnection suggests that H_2_O_2_ may not have acted primarily as a damaging ROS under these conditions, but rather as a signaling molecule involved in stress acclimation. Hydrogen peroxide is widely recognized as an important secondary messenger in plant stress responses, regulating antioxidant defenses, osmotic adjustment, and redox-dependent signaling pathways (Smirnoff and Arnaud, 2019; Dumanović et al., 2021). In this context, the maintenance of moderate H_2_O_2_ levels in NP GSNO-treated plants might have contributed to the activation of protective responses without inducing lipid peroxidation. Conversely, the reduction in H_2_O_2_ observed in untreated WD plants might reflect a broader suppression of ROS-producing metabolic processes under dehydration, potentially associated with reduced photosynthetic electron transport, or lower NADPH oxidase activity. Further analyses are necessary to substantiate these assumptions.

The maintenance of moderate H_2_O_2_ levels in NP GSNO-treated plants under WD, rather than a complete suppression or excessive accumulation of this ROS, is consistent with observations in other plant species, including sugarcane, maize, soybean, and *H. popayanensis* (Silveira et al., 2016, Silveira et al., 2017; Majeed et al., 2020; do Carmo et al., 2021; Astaneh et al., 2022; Rezayian et al., 2023). Moreover, the results of the present study corroborate previous findings that NO donors mitigate oxidative damage under abiotic stresses (Majeed et al., 2020; Astaneh et al., 2022; Shinde et al., 2024).

The conversion of a greater quantity of ROS, such as superoxide (O_2_^•−^), into molecules that are less reactive, including H_2_O_2_ and molecular oxygen (O_2_) is frequently facilitated by enzymatic antioxidants, including POD and SOD, and represents a pivotal step in ROS detoxification (Smirnoff and Arnaud, 2019; Dumanović et al., 2021). The antioxidant enzyme responses observed in the present study were strongly dependent on tissue type and water regime. In leaves, SOD activity was primarily affected by the water regime rather than by NP GSNO application itself, whereas POD activity increased in NP GSNO-treated plants only under WW conditions. In roots, NP GSNO promoted higher SOD activity under both water regimes, while POD activity was again more responsive under WW conditions. Therefore, the enzymatic responses observed here do not support a generalized stimulation of antioxidant enzymes under WD conditions. Instead, the reduction in lipid peroxidation observed in NP GSNO-treated plants under WD was more closely associated with the accumulation of non-enzymatic antioxidant metabolites and osmoprotective compounds. Indeed, under WD, NP GSNO-treated plants exhibited increased levels of polyols, flavonoids, catechin derivatives, and other stress-associated metabolites with recognized antioxidant properties. These compounds are known to contribute to ROS scavenging, membrane stabilization, and redox homeostasis under dehydration conditions (Bernatoniene and Kopustinskiene, 2018; Shomali et al., 2022).

The application of NP GSNO increased RSNO levels in leaves of *A. angustifolia*, indicating enhanced NO availability, since RSNOs are the main reservoir of NO in plants (Astier et al., 2018). Although the nanoparticles were applied to the substrate, RSNO accumulation occurred predominantly in leaves of WD plants. This pattern suggests that WD may favor systemic RSNO-mediated signaling in aerial tissues rather than local accumulation in roots. Such responses may result from stress-induced modulation of NO homeostasis, including changes in GSNOR activity, thioredoxins, and other redox-related components that regulate RSNO turnover (Majeed et al., 2020; Das et al., 2025; Rehaman et al., 2025). Because GSNO turnover is a key regulator of NO signaling and WD acclimation (Nabi et al., 2019; Khan et al., 2023), increased leaf RSNO levels may reflect a greater demand for redox regulation in photosynthetically active tissues. In contrast, the absence of RSNO accumulation in roots may indicate rapid local turnover or tighter control of NO homeostasis.

Besides GSNO, chitosan has also been demonstrated to contribute to stress mitigation by enhancing the antioxidant defense system (Ji et al., 2022; Shinde et al., 2024). Previous studies demonstrated that chitosan-based NPs can mitigate the deleterious effects of abiotic stresses (Behboudi et al., 2019; Ali et al., 2021). Additionally, while GSNO itself is a well-known antioxidant molecule that plays protective roles in plants under abiotic stresses (Saini et al., 2024; Perlikowski et al., 2025), the same can be said of the improved responses seen with the use of chitosan-based NPs (Silveira et al., 2019; do Carmo et al., 2021). Furthermore, the mucoadhesive properties of chitosan may have facilitated interactions between the nanoparticles and root surfaces, enhancing the GSNO effects (Seabra et al., 2014, Seabra et al., 2022).

The contribution of the NP carrier itself cannot be completely excluded from the responses observed. Although NP GSNO-treated plants exhibited more favorable morphological responses than NP GSH-treated plants, the protective effects likely resulted from interactions between GSNO signaling and the NP delivery system rather than from either component alone. Nevertheless, the superior growth performance of NP GSNO-treated plants suggests that GSNO was the main driver of the observed effects. Similar growth-promoting and stress-mitigating effects of NO donors have been reported in beetroot (*Beta vulgaris*) and soybean under adverse environmental conditions (Ekinci et al., 2020; Methela et al., 2023b). Interestingly, WD did not markedly reduce shoot length or shoot dry weight during the experimental period, even in control plants. This response may reflect the conservative growth strategy of conifer seedlings, which often maintain slow biomass accumulation under stress (Zlobin, 2022). Thus, the absence of pronounced growth reductions despite clear physiological and metabolic changes supports the resource-allocation framework proposed by Zlobin (2022).

On the other hand, root-related responses appeared to be more sensitive to the treatments applied. NP GSH-treated plants exhibited reduced root dry weight relative to control plants under both water regimes, suggesting that NP GSH alone may have altered redox balance or carbon allocation patterns, without triggering the protective NO-mediated signaling associated with NP GSNO. Although the mechanisms underlying this pattern remain unclear, the results indicate distinct effects of NP GSH and NP GSNO on root growth and further support the view that beneficial responses observed in NP GSNO-treated plants were more closely associated with NO release than with GSH or chitosan-based nanocarrier itself.

### Nanoencapsulated GSNO induces metabolic adjustments and osmoprotective responses associated with water deficit acclimation in *A. angustifolia*

Treatments involving GSNO have also been associated with adjustments in carbon metabolism and resource allocation in plants subjected to stress conditions (Kumar et al., 2019). In the present study, NP GSNO modulated soluble sugar content according to the water regime. Under WW conditions, NP GSNO treatment reduced TSS levels in both leaves and roots, suggesting that, in the absence of stress, GSNO signaling may alter carbon partitioning, reducing the pool of readily available soluble sugars when the demand for compatible solutes is low (Thalmann and Santelia, 2017).

In contrast, under WD, NP GSNO-treated plants exhibited relatively higher TSS levels than untreated plants, indicating that GSNO may help sustain soluble sugar availability during drought. Soluble sugars play central roles in osmotic adjustment, stabilization of cellular structures, and maintenance of metabolism under dehydration (Jeandet et al., 2022; Saini et al., 2024). Consistently, WD plants treated with NP GSNO also showed increased levels of key osmoprotectants, including proline, sucrose and trehalose, as well as higher glucose accumulation, which may serve as an accessible energy source to support stress-responsive metabolism and cellular repair processes (Siddiqui et al., 2020; Akin and Kaya, 2024; Eh et al., 2024). Together, these results indicate that NP GSNO promotes a context-dependent metabolic response, reducing soluble sugar pool under optimal water supply while maintaining enhanced soluble sugar and osmoprotectant accumulation under water deficit.

This osmotic adjustment was accompanied by broader metabolic changes involving amino acids and N metabolism. Increased glutamine levels in leaves suggest that NP GSNO may promote N storage in the form of amides, potentially contributing to the maintenance of N metabolism under stress. This interpretation is supported by previous evidence that GSNO can protect glutamine synthetase (GS) activity and regulate glutamine and glutamate pools, which serve as precursors for osmoprotectant synthesis and provide carbon skeletons for sustained growth under dehydration (Yin et al., 2022). Likewise, the accumulation of asparagine in roots under WD and NP GSNO-treated WD conditions reinforces the role of N-rich compounds in osmoprotection and metabolic buffering during stress.

In addition to sugars and amino acids, NP GSNO treatment increased the accumulation of polyols, suggesting an important role for these compounds in tissue-specific osmoprotection. Osmoprotection in roots under NP GSNO-treated WD appeared to rely more heavily on polyol-mediated mechanisms than in leaves. Although literature evidence linking NP GSNO to the specific induction of polyols remains limited, these compounds derive from central carbon metabolism and may therefore be indirectly enhanced by GSNO-driven stress-associated metabolic responses. Studies have indicated that the presence of mannitol helps to decrease water potential while increasing cellular osmotic pressure, thereby maintaining turgor and reducing water loss. Furthermore, mannitol and other polyols can mimic water molecules, forming an artificial hydration shell around macromolecules (Dichio et al., 2009; Edmond et al., 2025). Similarly, myo-inositol plays a dual role, acting both as a metabolic intermediate in plants and as a key contributor to soil nutrient cycling. Roots that absorb and accumulate myo-inositol exhibit enhanced intracellular signaling and increased phosphate uptake. This coordinated response helps plants to effectively cope with nutrient limitation and WD (Su and Saiardi, 2024). Thus, the accumulation of mannitol and myo-inositol in *A*. *angustifolia* roots may have contributed not only to osmotic adjustment and cellular protection but also to signaling process associated with stress acclimation.

Beyond osmotic regulation, NP GSNO also enhanced antioxidant defenses, through the accumulation of secondary metabolites. Increased levels of flavonoids, particularly catechin derivatives, highlight their contribution to stress mitigation via ROS and RNS scavenging (Bernatoniene and Kopustinskiene, 2018; Shomali et al., 2022). In parallel, changes in metabolites associated with the shikimic acid pathway, indicated by elevated quinic and shikimic acids, together with increased catechin and epigallocatechin accumulation, further supports the reinforcement of antioxidant capacity under WD conditions. These compounds are known not only for their strong antioxidant activity but also for their involvement in stress-related signaling pathways, including interactions with ABA-mediated responses (Baozhu et al., 2022; Nayak et al., 2025).

Polyols also contribute to antioxidant defense, either directly, as in the case of mannitol acting as a hydroxyl radical scavenger, or indirectly through myo-inositol-derived pathways linked to redox homeostasis and the ascorbate–glutathione cycle (Fatima et al., 2024; Edmond et al., 2025; Liu et al., 2025). The coordinated increase in these antioxidant metabolites suggests that NP GSNO may enhance the capacity of plants to mitigate oxidative and nitrosative stress under water deficit.

Importantly, by contributing to redox homeostasis, these metabolites may also help preserve GSNO stability under conditions of elevated ROS, ensuring maintenance of GSNO-dependent signaling pathways that regulate key adaptive responses, including osmotic adjustment and root growth (Lau et al., 2021; Akhtar et al., 2026). Taken together, these adjustments suggest that the protective effects of NP GSNO under WD extended beyond antioxidant responses, probably involving broader signaling networks that coordinate stress acclimation. Recently, Gul et al. (2026) showed that the interplay among ROS, RNS, and salicylic acid promotes antioxidant activation, osmoprotectant accumulation, and hormonal signaling during seedling establishment. Thud, the accumulation of osmoprotective metabolites and the modulation of redox-associated compounds in NP GSNO-treated *A. angustifolia* seedlings may reflect integrated NO-mediated hormonal and metabolic acclimation responses under WD.

Overall, the results observed in the present study suggest that NP GSNO influences long-term metabolic adjustments associated with carbon allocation, osmoprotectant accumulation, and reinforcement of antioxidant defenses, collectively contributing to improved WD tolerance in both leaves and roots of *A. angustifolia*. Nevertheless, because all analyses were performed only at the end of the experimental period, the temporal dynamics of NP GSNO-mediated responses under WD remain unresolved. Consequently, the metabolic changes observed here likely reflect late-stage acclimation responses rather than early signaling events. Future studies incorporating intermediate sampling points and time-course analyses are important to better characterize the progression of NP GSNO-mediated WD responses and the temporal interplay between ROS/RNS signaling and metabolic adjustments in *A. angustifolia* seedlings.

### Water deficit induced metabolic adjustments associated with structural stabilization, energy supply and root growth

The simultaneous increase in malic acid in leaves and in lipid metabolism-related compounds in both leaves and roots of *A*. *angustifolia* suggests a metabolic reprogramming aimed at maintaining cellular homeostasis under WD conditions. Malate accumulation may be associated with osmotic adjustment and the metabolic homeostasis (Zhao et al., 2025). As a central intermediate of carbon metabolism, malate integrates multiple metabolic pathways, contributing to carbon redistribution and cellular redox balance (Zhao et al., 2025). Concurrently, lipids may contribute to maintain membrane stability and serve as energy reserves to support cellular metabolism under stress (Cheng et al., 2026).

Under WD treatments, the accumulation of palmitic and heptadecanoic acids suggests adjusts in lipid metabolism associated with membrane stability. A higher proportion of saturated fatty acids might contribute to reduced membrane permeability and increased resistance of the lipid bilayer to stress-induced damage, thereby supporting the maintenance of cellular integrity (Upchurch, 2008; Vieira et al., 2022). Moreover, palmitic and stearic acids serve as precursors for very-long-chain fatty acids, which are components of cuticular waxes involved in limiting non-stomatal water loss under WD conditions (Lee and Suh, 2015). Similarly, the fatty acid-derived amides 1-hexadecanamide, myristamide and dodecanamide, which accumulated exclusively in roots under WD conditions, may represent additional components of lipid-related stress responses. Although their roles in *A. angustifolia* remain unclear, fatty acid amides have been associated with stress signaling and lipid metabolism in plants (Hou et al., 2016). Their accumulation, together with changes in fatty acid profiles, suggests adjustments in lipid-related pathways that may contribute to cellular homeostasis under WD.

Consistent with this, NP GSNO treatment was associated with reduced oxidative stress and lipid peroxidation in both roots and leaves of *A. angustifolia*, indicating preservation of membrane integrity and the cellular lipid pool (Lei et al., 2025; Gao et al., 2026). This effect may indirectly favor the maintenance of lipid-derived compounds, including fatty acid amides. Altogether, the integration of oxidative stress protection and structural damage stabilization appears to be a key mechanism underlying WD tolerance in *A. angustifolia*.

### Ecophysiological significance and applications for conservation and reforestation

The findings of the current study indicate that nanoencapsulated GSNO was associated with modulation of antioxidant responses and attenuation of oxidative damage in plants subjected to WD. In addition to enhancing enzymatic antioxidant activity, nanoencapsulated GSNO modulated non-enzymatic antioxidants, offering further protection against the detrimental impacts of WD stress. These protective effects align with findings from previous research, including studies on Neotropical tree species (do Carmo et al., 2021, do Carmo et al., 2025). Interestingly, the concentration used in the present study was fivefold higher than that reported by do Carmo et al. 2021, do Carmo et al., 2025). Previous studies from our group have shown that this concentration appears to be supraoptimal for several native tree species, leading to disruptions in NO homeostasis and consequent deleterious effects. Given that ecophysiological studies on gymnosperms, and particularly on the genus *Araucaria*, remain scarce, these findings highlight the importance of further investigations on GSNO signaling and stress responses in this lineage.

From an applied perspective, integrating nanotechnology-based approaches with traditional reforestation practices may offer new tools to improve seedling performance under increasingly frequent drought events. While further studies are needed to assess long-term responses and optimal dosages, the use of GSNO-loaded chitosan NPs emerges as a promising strategy to enhance stress resilience during the critical early stages of tree establishment. Expanding this approach to other tree species may contribute to the development of innovative conservation and restoration practices aimed at mitigating the impacts of climate change on forest ecosystems. However, despite the promising physiological and metabolic responses observed in the present study, the potential application of nanoencapsulated GSNO in conservation and reforestation programs should be interpreted with caution. The experiments were conducted under controlled greenhouse conditions using young seedlings and a single substrate application, which may not fully represent the complexity and environmental variability encountered under field conditions.

Factors such as nanoparticle stability, NP GSNO persistence in the substrate, release dynamics under different soil physicochemical conditions, and long-term interactions with soil microbiota may substantially influence the efficacy and environmental behavior of the formulation in natural ecosystems. It is noteworthy that chitosan-based nanoparticles are generally considered biodegradable and biocompatible, raising few environmental concerns (Karimlar et al., 2026). Before practical implementation in restoration and conservation programs can be realistically considered, it is essential that further studies be performed, that include long-term experiments, repeated applications, and field-based validation.

## Data Availability

The raw data supporting the conclusions of this article will be made available by the authors, without undue reservation.
