# Supplementary material for: S-nitrosoglutathione-loaded chitosan nanoparticles promote adaptive responses to water deficit in the critically endangered conifer Araucaria angustifolia
Source: Front Plant Sci. 2026 Jul 15;17:1864816. doi: 10.3389/fpls.2026.1864816 (PMC13414298; doi:10.3389/fpls.2026.1864816)
Supplement: Supplementary file 1 [file Supplementaryfile1.pdf]

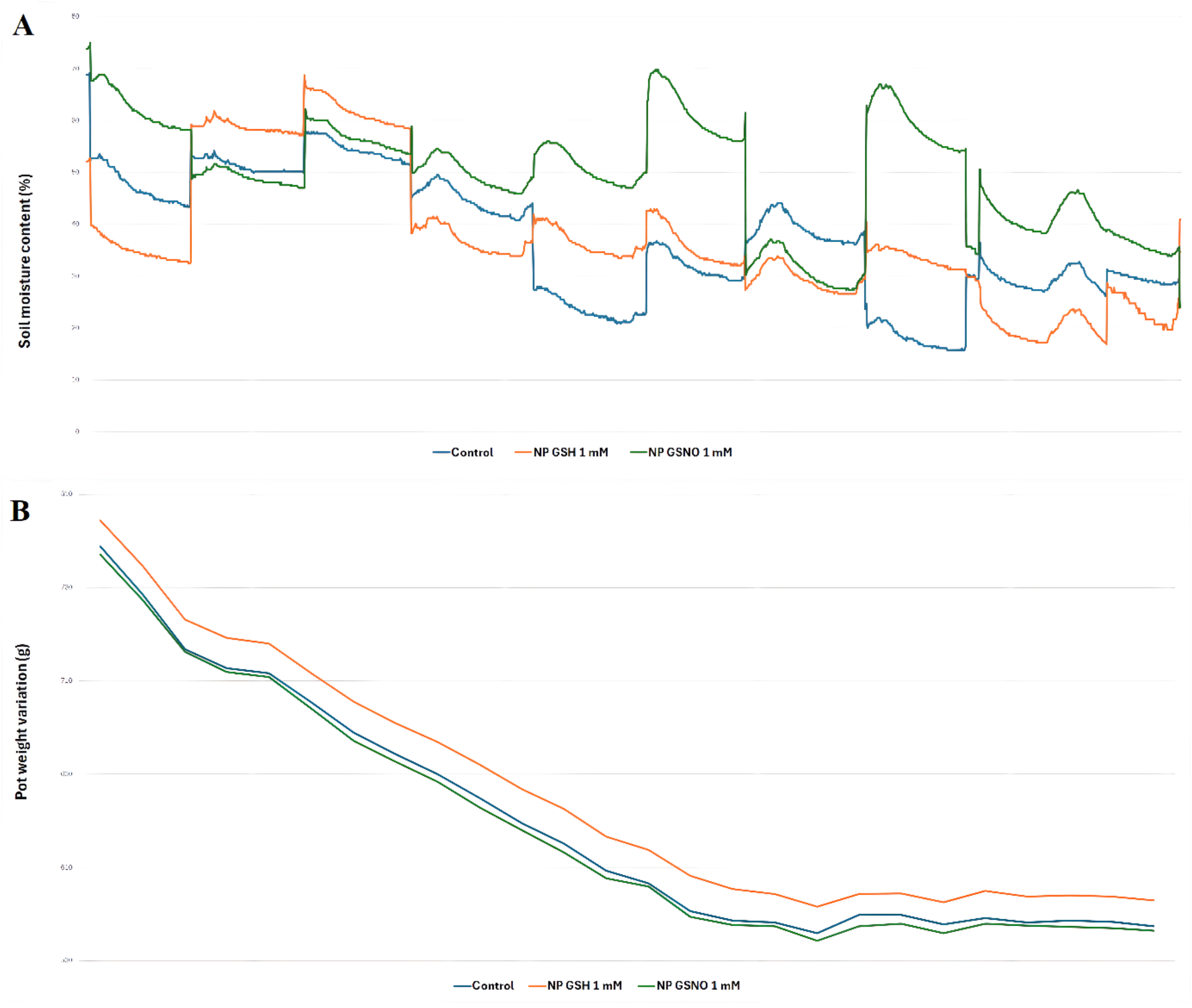

Figure S1. (A) Daily variation in soil moisture content (%) throughout the experimental period (30 days) with *A. angustifolia* in control, NP GSH 1 mM, and NP GSNO 1 mM treatments under WD conditions. Vertical drops indicate irrigation intervals used to maintain the target soil moisture. (B) Pot weight variation (g) measured during the progression of WD. Values represent the mean of biological replicates.

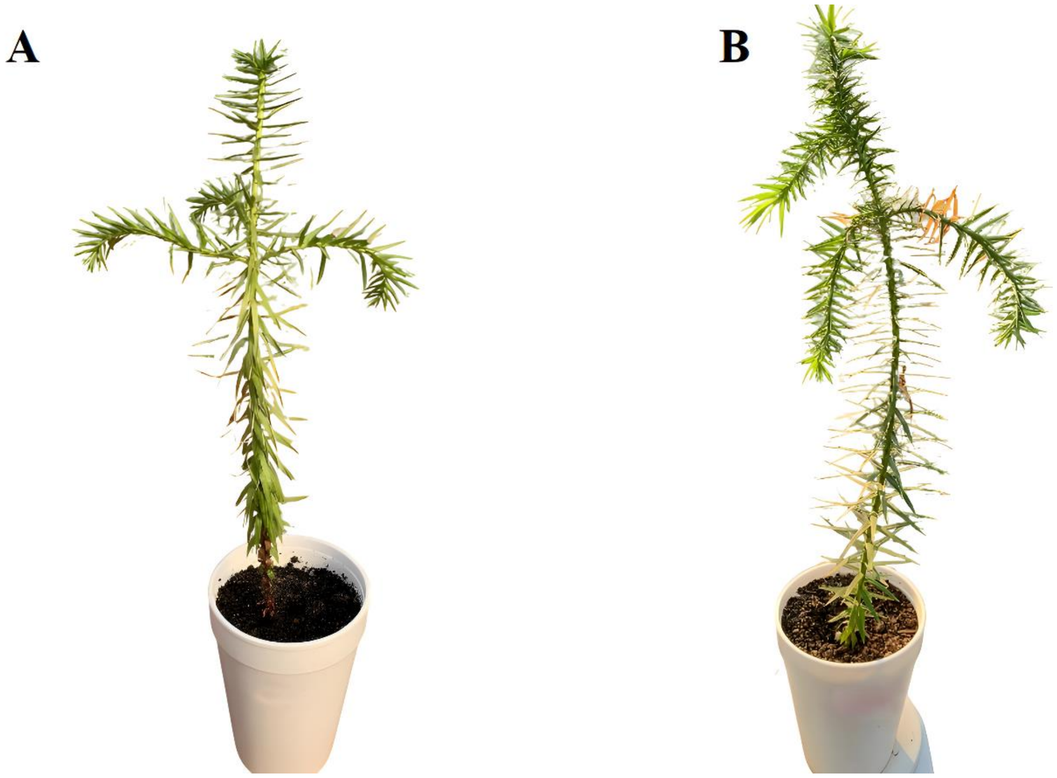

Figure S2. Representative image of the leaves of *A. angustifolia* seedlings illustrating the needle-like morphology characteristic of the species, which limits the use of conventional gas exchange chambers commonly employed for broadleaf species.

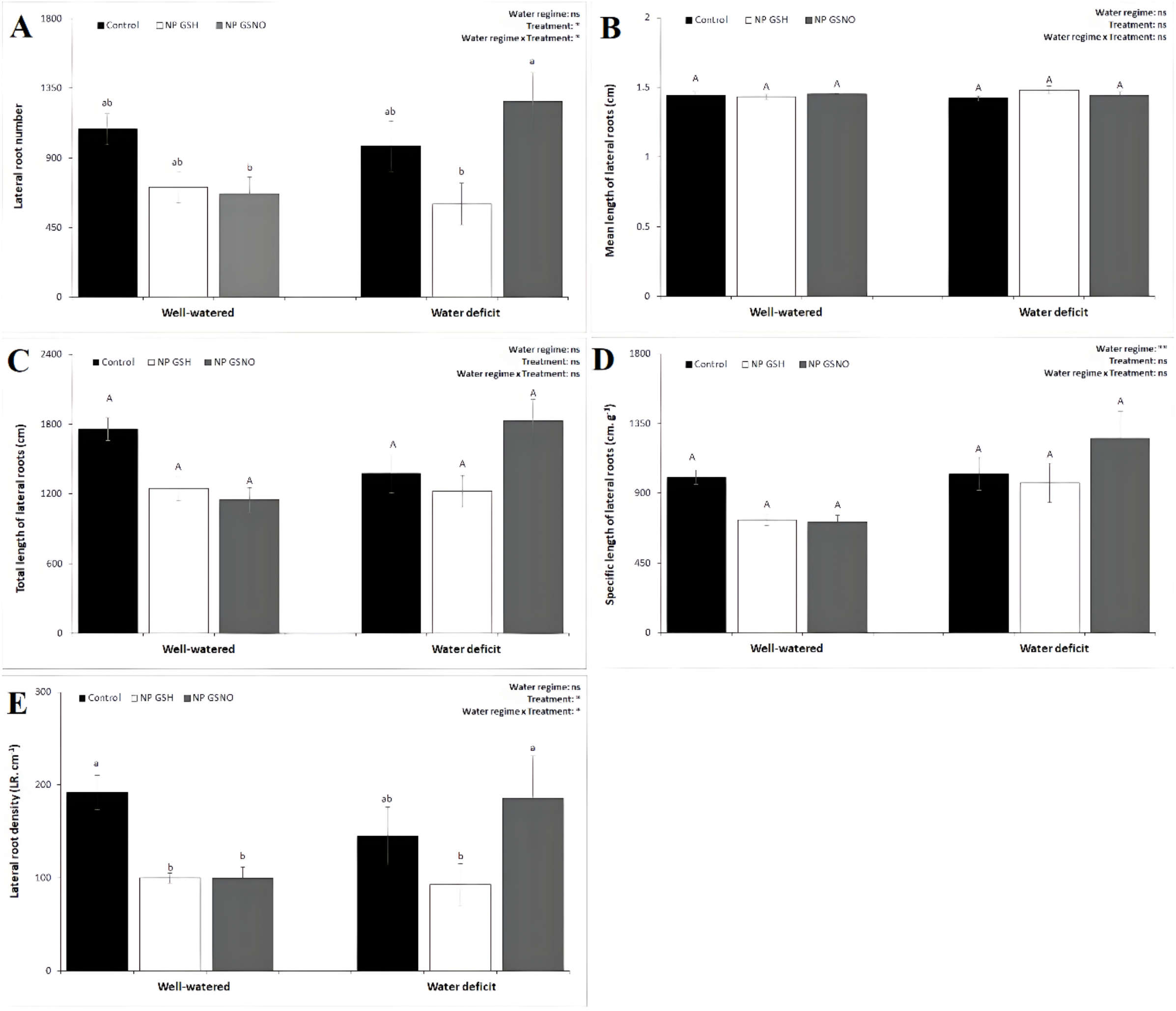

Figure S3. Root architectural traits of *A. angustifolia* seedlings subjected to well-watered (WW) and water deficit (WD) conditions and treated with nanoencapsulated GSH and nanoencapsulated GSNO (both at 1 mM). (A) lateral root number, (B) mean length of lateral roots, (C) total length of lateral roots, (D) specific length of lateral roots, (E) lateral root density. Values represent means ± standard-error. Plants were grown under well-watered or water-deficit conditions and treated with NPs containing GSH (white bars), GSNO (gray bars), or water-only as a control (black bars). Values are means ± standard error (SE). Different lowercase letters indicate significant differences among treatment × water-regime combinations, while different uppercase letters indicate significant main effects of treatment within each water regime, as determined by two-way ANOVA followed by post hoc tests ($p < 0.05$). Significance of main effects and interactions is denoted as *** $p < 0.001$, ** $p < 0.01$, * $p < 0.05$, and ns (not significant).

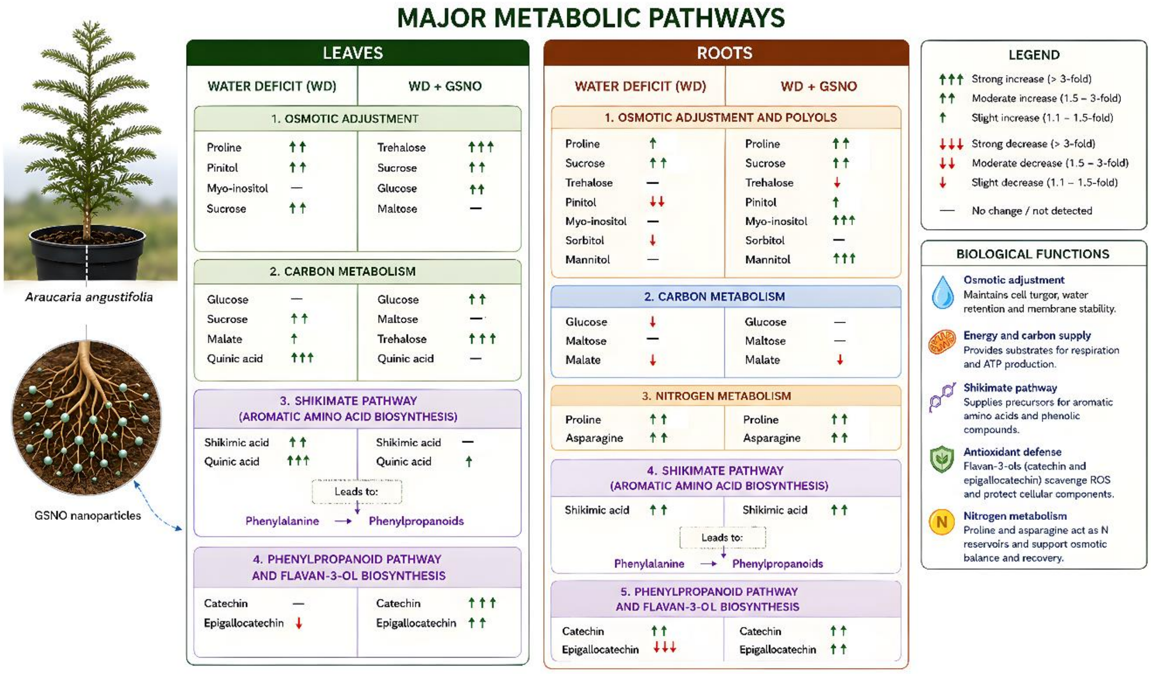

Figure S4. Schematic overview of the principal metabolic adjustments induced by water deficit (WD) and NP GSNO treatment in leaves and roots of *A. angustifolia* seedlings. Metabolic responses are grouped into five major functional categories: osmotic adjustment, carbon metabolism, nitrogen metabolism, shikimate pathway, and phenylpropanoid/flavan biosynthesis. Arrows indicate the direction and relative magnitude of metabolite accumulation compared with well-watered control plants. The figure highlights tissue-specific metabolic reprogramming associated with osmoprotection, antioxidant defense, carbon and nitrogen metabolism, and stress acclimation under WD conditions. This figure was partially created using artificial intelligence-assisted graphic design tools and subsequently modified and validated by the authors.
