# Supplementary material for: S-nitrosoglutathione-loaded chitosan nanoparticles promote adaptive responses to water deficit in the critically endangered conifer Araucaria angustifolia
Source: Front Plant Sci. 2026 Jul 15;17:1864816. doi: 10.3389/fpls.2026.1864816 (PMC13414298; doi:10.3389/fpls.2026.1864816)
Supplement: Supplementary file 2 [file Table1.pdf]

Table S1. Morphological and root architectural parameters of *A. angustifolia* seedlings subjected to different water regimes and treated with nanoencapsulated GSNO. Shoot length (SL), primary root length (PRL), shoot dry weight (SDW), hypocotyl dry weight (HDW), root dry weight (RDW), lateral root number (LRN), total root length (TLR), specific root length (SLR), total length of lateral roots (TLLR), median length of lateral roots (MLLR), specific length of lateral roots (SLLR), and lateral root density (LRD) were evaluated after 30 days under well-watered (WW) and water deficit (WD) conditions. Values represent mean ± standard error (n = 12). Uppercase and lowercase letters indicate significant differences among water regime and treatments, respectively. Different lowercase letters combined with a symbol (*) indicate a statistically significant difference resulting from the interaction, i.e., the combined effect of the two variables, water regime and treatment (Two-way ANOVA, Tukey's HSD, $p < 0.05$).

Treatment	Water regime	SL	PRL	SDW	HDW	RDW	LRN	TLR	SLR	TLLR	MLLR	SLLR	LRD
Control	WW	42.7 ± 1.75 bc*	6.44 ± 0.341	8.37 ± 0.257 a*	0.454 ± 0.054	1.7 ± 0.118 a*	1085 ± 99.1 ab*	1751 ± 177 a*	1112 ± 41.6 ab*	1761 ± 164 ab*	1.44 ± 0.03	1003 ± 46.9 ab*	192 ± 18.7
NP GSNO 1	WW	44 ± 0.619 bc*	5.96 ± 0.04	7.55 ± 0.468 ab*	0.325 ± 0.024	1.22 ± 0.11 bc*	667 ± 108 ab*	975 ± 163 ab*	720 ± 43.9 b*	1148 ± 152 ab*	1.45 ± 0.006	715 ± 44.1 b*	99.1 ± 12.9
NP GSNO 10	WW	41.1 ± 1.87 c*	7.13 ± 1.09	6.93 ± 0.566 ab*	0.448 ± 0.036	1.46 ± 0.102 ab*	1230 ± 167 a*	1511 ± 219 ab*	1203 ± 56.4 a*	1988 ± 229 a*	1.45 ± 0.026	1193 ± 57.4 a*	126 ± 28.5
Control	WD	43.4 ± 1.6 bc*	6.12 ± 0.097	5.99 ± 0.725 b*	0.38 ± 0.021	1.52 ± 0.128 ab*	975 ± 165 ab*	1382 ± 228 ab*	1030 ± 107 ab*	1375 ± 228 ab*	1.42 ± 0.016	1024 ± 107 ab*	145 ± 31.3
NP GSNO 1	WD	48.2 ± 1.42 ab*	6.58 ± 0.139	7.86 ± 0.48 ab*	0.473 ± 0.041	1.46 ± 0.051 ab*	1264 ± 186 a*	1839 ± 272 a*	1259 ± 175 a*	1830 ± 273 ab*	1.44 ± 0.032	1253 ± 176 a*	186 ± 45.6
NP GSNO 10	WD	50.9 ± 1.44 a*	6.2 ± 0.207	6.54 ± 0.396 ab*	0.362 ± 0.02	1 ± 0.043 c*	545 ± 23.5 b*	617 ± 181 b*	1128 ± 160 ab*	1073 ± 173 b*	1.22 ± 0.216	1122 ± 160 ab*	103 ± 12.4
**Anova two-way**													
Treatment		ns	ns	ns	ns	**	ns	ns	ns	ns	ns	ns	ns
Water regime		***	ns	*	ns	ns	ns	ns	ns	ns	ns	ns	ns
Treatment x Water regime		*	ns	*	ns	**	**	**	*	**	ns	*	ns
